# Do parent media habits contribute to child global development?

**DOI:** 10.3389/fpsyg.2023.1279893

**Published:** 2024-01-10

**Authors:** Caroline Fitzpatrick, Alexa Johnson, Angélique Laurent, Mathieu Bégin, Elizabeth Harvey

**Affiliations:** ^1^Department of Preschool and Elementary School Education, Université de Sherbrooke, Sherbrooke, QC, Canada; ^2^Department of Childhood Education, University of Johannesburg, Johannesburg, South Africa; ^3^Département des Sciences de l’Éducation, Université Sainte-Anne, Church Point, NS, Canada

**Keywords:** parent media use, parent screen use, global development, ages and stages questionnaire, preschool, early childhood

## Abstract

**Background/objective:**

Parents of preschoolers’ report using screen media frequently. More frequent screen use by parents may undermine child development by displacing time for foundational parent-child interactions. The objective of the present study is to examine the extent to which parent screen use contributes to child global development 1 year later.

**Methods:**

Data are from a cohort of 315 preschoolers from Nova Scotia, Canada and their parents during the COVID-19 pandemic. Parents reported the number of hours per day they spent using screens, as well as child screen time and sex, and parent educational attainment. Our outcome is child global development scores, which combine assessments of communication, cognitive, personal-social, and motor skills measured at 4.5 using the *Ages and Stages Questionnaire* (ASQ) (*N* = 249, 79% retained).

**Results:**

Parents in our sample spent on average 6.35 h per day using screen media outside of work (SD = 3.07) and children spent on average 3.43 h per/day using screens. Multivariate linear regression indicated that each 1-h increase in parents daily screen media use, corresponded to a 1.25 decrease in child global development scores, *B* = −1.25 *p* < 0.05, 95% CI between −2.37 and −0.13.

**Conclusion:**

Our results indicate that parent screen use may represent a key component of children’s media ecology. Given the importance of global development in early childhood for later health and achievement, the present results suggest that interventions should include parent screen use habits in media wellness interventions.

## 1 Introduction

Screen media, referring to content that is available through electronic devices such as televisions, computers, phones, and tablets, has infiltrated young children’s personal and family ecologies (Rideout and Robb, 2020). The impact of accumulating too much screen time by preschool-aged children on their health and development have been previously demonstrated with studies indicating that child screen time can undermine their brain development, sleep, and fitness (Pagani et al., 2010; Jones et al., 2013; Lan et al., 2020). Research also suggests that screen time can undermine global child development across motor, communication, cognitive, and personal/social domains, and increase risks for developmental delays (Madigan et al., 2019). Global child development is a key predictor of school readiness and early achievement (Prior et al., 2011; Józsa et al., 2022). Research has found that one out of every four child is at risk of beginning kindergarten without the requisite skills set to success (Browne et al., 2018). Given the omnipresence of screens in children’s ecology, better understanding how screens in children’s early environments contribute to global development remains crucial.

According to ecological theories of child development, parental screen time is also of concern (Barr, 2019). One study conducted prior to the pandemic found that mothers of 3-year-olds spend 3 h per day using screen media (Madigan et al., 2020). More recently, during the COVID-19 lockdowns, parents of preschoolers reported spending as many as 6 h per day of personal screen time (Fitzpatrick et al., 2022). This may be the case because the pandemic and its accompanying confinement measures led many families to become more reliant on screens for daily activities and socializing.

According to one review, parent screen time can distract parents and lead them to be less verbally and non-verbally responsive to their child (Kildare and Middlemiss, 2017). Furthermore, another review suggests that parent mobile devise use can reduce parent sensitivity toward their child (Braune-Krickau et al., 2021). As such, to more fully understand how children’s media ecology is shaping their development, it remains important to also consider whether parent screen use may have an impact on young children’s development.

Early childhood represents a key developmental window for acquiring key cognitive, motor, and social skills. Young children, in particular, depend on sensitive, warm, and reciprocal interactions with caregivers to acquire foundations skills across these domains. Existing research has examined how parent mobile device use and attitudes toward technology relate to child media habits (Cingel and Krcmar, 2013; Lauricella et al., 2015; Pila et al., 2021). However, to date, few studies have examined how parent screen use contributes to children’s later developmental skills. One exception is a longitudinal study that followed parents of children ages 1–5 and found that parental distraction with technology is associated with an increased risk of children developing behavioral problems, including emotional reactivity, tantrums, withdrawal, and anxiety (McDaniel and Radesky, 2018). Research has yet to examine how parent screen time may influence global child development across motor, social, and cognitive domains.

The COVID-19 pandemic was accompanied by increases in family screen media use (Hartshorne et al., 2021). As such, it remains useful to examine parent screen use habits and their consequences during this challenging time. In addition, most research on this topic has been cross-sectional and conducted with infants and toddlers. As such, little is known about the potential impact of parental screen use on children during the preschool years. Finally, previous research has found that family screen use and child global development are associated with child and family characteristics including child sex and screen time, socioeconomic status, and access to financial and personal resources (Dohnt and Tiggemann, 2006; Cingel and Krcmar, 2013; Lauricella et al., 2015; Kildare and Middlemiss, 2017; McDaniel and Radesky, 2018; Madigan et al., 2020; Braune-Krickau et al., 2021; Hartshorne et al., 2021; Pila et al., 2021; Fitzpatrick et al., 2022; Rideout et al., 2022). Thus, the objective of this longitudinal study is to better understand if parental screen use is associated with preschooler global development. We hypothesize that greater parent screen use will forecast lower child global development scores. To better isolate the potential contribution of parent screen use, we estimate associations while statistically controlling for child sex and baseline screen time and parent education.

## 2 Materials and methods

### 2.1 Sample and procedure

This 2-year longitudinal study followed parents (*N* = 315) and their preschool-aged children (mean age = 3.46, age range between 2 and 5). The baseline data collection took place between April and August 2020, during the first wave of the COVID-19 pandemic and a provincially declared state of emergency, in Nova Scotia, Canada. Participants were recruited using multiple strategies including through posters and pamphlets distributed in daycares and schools, and family clinics, as well as through advertisements in the newspapers and broadcast on the radio across Nova Scotia. Mothers were the respondents in 94% of cases. Most participants reported they were married (82%), born in Canada (91%), and white (90.5%). Of the sample, 53% of the children were male (*N* = 168) and 47% were female (*N* = 147). The majority of participants reported that English was the most spoken language in their home (88.1%). To measure the child and parent screen time, participants were asked to complete an online questionnaire when children were 3.5 years old. One year later, when children were 4.5 years old, parents rated child global development (*N* = 249,79% retained) remotely. Parents were compensated for their time with a 50$ gift certificate at each data collection. Parents also provided informed consent to participate at each wave of the study. This project received ethics approval from Université Sainte-Anne and Université de Sherbrooke’s internal review boards.

### 2.2 Measures: outcomes

Parents completed the Ages and Stages Questionnaire third edition (Squires et al., 2009) to assess 5 areas of child development: Communication; Fine motor skills; Gross motor skills; Problem-solving; and Personal/social development. In total, parents answered 30 statements (6 questions per domain of development) on their child’s ability to perform a task. Response options were: (1) Yes, scored as 2; (2) Sometimes, scored as 1; or (3) Not yet, scored as 0. A global development score was computed by summing the scores across all domains of development. The ASQ screening tool is routinely used in clinical settings to screen for developmental delays (Richter and Janson, 2007). The validity, sensitivity, reliability, and specificity of this scale have been demonstrated in several studies (Gollenberg et al., 2010; Schonhaut et al., 2013; Singh et al., 2016). To account for the range in child age in our sample, we computed age-adjusted scores by subtracting the age normed clinical cut-off from each child’s score. As such, negative scores reflect that a child was below their clinical cut-off whereas a positive score indicates that a child was above their clinical cut-off.

### 2.3 Measures: main predictor

Parents completed the Media Assessment Questionnaire (MAQ) (Barr et al., 2020) to provide estimates of the amount of time they spent engaged with the following devices on weekdays and weekend days separately, outside of work hours: TV/DVD, computer, video games consoles, iPad, tablet, and smartphone. Response options included: (1) Never; (2) Less than 30 min; (3) 30 min to 1 h; (4) 1–2 h; (5) 2–3 h; (6) 4–5 h; (7) more than 5 h. We then converted these categorical responses into continuous variables reflecting the number of hours spent with each type of media. Our approach involved using the midpoint for each response range, with the exception of “5 or more hours a day” where a more conservative score of 5 was used. Daily weighted estimates were then estimated by multiplying weekday estimates by 5 and weekend day estimates by 2 and dividing the total by 7. Finally, we calculated an overall daily screen time estimate by summing average daily usage across media devices.

### 2.4 Control variables

Parents also reported child media use using the MAQ. More specifically, parents reported the average amount of time children spent doing each of the following on weekdays and weekend days separately: (1) watching TV or DVDs; (2) using a computer; (3) playing video games on a console; (4); Using an iPad, tablet, LeapPad, iTouch, or similar mobile device (excluding smartphones); or (5) Using a smartphone. Response options included: (1) Never; (2) Less than 30 min; (3) 30 min to 1 h; (4) 1–2 h; (5) 2–3 h; (6) 4–5 h; and (7) more than 5 h. We then used the same approach as used with the parent screen time measure to create a weighted daily estimate of child screen time. Finally, we calculated an overall daily screen time estimate by summing average daily usage across media devices. Parents also reported child sex, and their educational attainment which was dichotomized as either: (1) High school or college vocational (*N* = 81); and (2) University degree (*N* = 234).

## 3 Results

### 3.1 Descriptive and bivariate statistics

Descriptive statistics and frequencies are presented in Tables 1, 2, respectively. Table 3 shows bivariate associations between parent and child screen time and later child global development. Parents and children in our sample spent on average 6.35 (SD = 3.07) and 3.43 (SD = 2.44) hours per day using screens, respectively. Girls performed better than boys on the assessment of global development (mean = 120.52 vs. 105.62). In general, there were very few children that did not meet the global development clinical cut-off (1.2%). Overall, for each domain few children met the clinical cut-off for developmental delays in gross (4.8%) and fine (4.4%) motor, personal/social (4.4%), communication (2.4%), and problem solving (1.2%) domains of development. Bivariate correlations indicated that parent screen time (*r* = −0.18, *p* < 0.01) and child screen time (*r* = −0.13, *p* < 0.01) were both significantly negatively correlated with child global development scores. Furthermore, in terms of the sub-domains of global child development, parent screen time was significantly negatively associated with child communication (*r* = −0.15, *p* < 0.05), gross motor development (*r* = −0.13, *p* < 0.05), and problem solving (*r* = −0.18, *p* < 0.01).

**TABLE 1:** Descriptive statistics for continuous study variables.

	Mean (SD)	*N*	*N* (% missing)
**Age 3**			
Parent screen time	6.35 (3.08)	315	0
Child screen time	3.43 (2.44)	315	0
**Age 4**			
Global development	112.54 (32.48)	250	66 (21%)

**TABLE 2:** Frequencies and proportions for categorical variables.

	*N* (%)	*N* (% missing)
Parent educational attainment		0
High school/college	81 (25.60%)	
University degree	234 (74.10%)	
Child sex		0
Male	168 (53.20%)	
Female	146 (46.20%)	

**TABLE 3:** Bivariate correlations between parent media use and child developmental outcomes.

	1	2	3
**Predictors**			
1. Parent ST	–	0.45***	−0.18**
2. Child ST			−0.13**
**Outcomes**			
3. Global development		–	–

ST, screen time.

**p > 0.01,

***p > 0.001.

### 3.2 Missing data

In total 79% of our sample had complete data at both assessments when they were 3.5 and 4.5. Children with parents with a university degree were more likely than those without to remain in our sample at the second wave, χ (12) = 5.37 *p* = 0.020. Child sex and screen time were unrelated to participant attrition. Little’s test conducted in SPSS was non-significant, which provides evidence that our data was MCAR, χ (12) = 16.43, *P* = 0.172. As such, following best practices for treating missing data, we estimated 5 imputed data sets using the multiple imputation function in SPSS and conduct our analyses over these pooled estimates (Cummings, 2013).

### 3.3 Multiple regression analyses

Regression results are presented in Table 4. An adjusted multiple regression was estimated to measure associations between parent screen use when children were 3.5 and global child development when the child was 4.5. Child’s own screen time, sex, and parent education were controlled. Analyses revealed that each 1-h increase in parents daily screen use corresponded to a 1.25 decrease in child global development scores, (*B* = −1.25 *p* < 0.05, 95% CI between −2.37 and −0.13). Practically, our results suggest that an average of 6 h of parental screen time daily would correspond to 1.86 and 7.5 score reductions global development scores. As such, associations indicate that parental screen time could account for decreases in approximately 23% of a standard deviation.

**TABLE 4:** Adjusted unstandardized regression coefficients estimating the contribution of parents and child screen time to global child development.

	Global development	
	**B (95% CI)**	***P*-Value**
Parent screen time	−1.25 (−2.37 τo−0.13)	0.029
Child screen time	−1.05 (0.67 τo 0.01)	0.293
**Child sex**		
Girl	14.71 (8.51 to -20.93)	<0.001
Boy (reference)	–	–
**Parent education**		
Bachelors/graduate	−1.58 (−8.84 τo 5.69)	0.670
HS/vocational	–	–
R square	0.09	

Results are adjusted for child screen time, child sex, and parent education.

## 4 Discussion

In the present study, we examine whether parental screen time when children were 3.5 was predictive of later global child development at age 4.5. In support of our hypothesis, we found that the number of hours parents spent using screens was associated with lower child global development scores 1 year later. Each of these in turn is considered a key determinant of children’s ability to successfully transition to and benefit form, school-based learning at the time of kindergarten entry (Duncan et al., 2007; Grissmer et al., 2010).

Our research adds to the literature by suggesting that more frequent and lengthy parent screen use may represent a risk factor for poorer developmental outcomes in preschoolers. According to ecological theories of development, learning in the early years is highly dependent on the social environment of the child and in particular, their interactions within their microsystems (Barr, 2019). The intensive use of screen media by parents is likely to interfere with the timing of these interactions by occasioning distractions. For instance, according to one study of mothers with children aged 3 or less, screens had disrupted parent-child interactions for 65% of the sample during playtime, 36% during book reading, 26% during mealtime, 26% during bedtime, and 22% while setting limits or disciplining the child (McDaniel and Coyne, 2016).

Similarly, our findings are consistent with the *displacement hypothesis*. The preschool years are crucial for experience-dependent learning. Accordingly, too much time devoted to screens by parents may limit the amount of time they are able to allocate to enriching activities that could help support child global development across physical, cognitive, and social domains.

Studies have also found that screen use by parents may reduce the amount of learning support provided to children. More specifically, parental mobile device use may interfere with scaffolding (i.e., providing timely feedback to the child), joint attention (ex. coordinating attention on the same object as the child), directiveness (ex., providing verbal and non-verbal directives to the child) (Corkin et al., 2021; Ochoa et al., 2021). Furthermore, according to the same studies, parental mobile device use was associated with lower child vocabulary. In line with these results, another observational study found that mothers who spontaneously used their mobile devices during a structured laboratory task initiated less verbal and non-verbal interactions with their preschool-aged child (Konrad et al., 2021).

In addition to disrupting dyadic parent-child interactions, parents also report that screens including cell phones/smartphones, television, computers/laptops, and iPads or other tablets interfere with coparenting, especially during activities like child play (McDaniel and Coyne, 2016). According to the same study, mothers who reported more interference from screens reported worse relationship satisfaction with their partners, and higher levels of depressive symptoms. As such, future research could seek to clarify the role that parental relationship quality and mental health may play in the association between parent screen use and child global development.

The present study presents some strengths. First, our study is the first to examine links between parent screen use outside of work and later global development in preschoolers. Furthermore, our study allows us to shed light on these association using a prospective study design implemented during the COVID-19 pandemic.

In terms of limits, our study was conducted remotely, due to public health measures in place at the time of data collection. As such, it was not possible to directly observe parent’s use of screens and account for extent to which parents might have been using screens in the presence of their child, and the extent to which parental screen use may have been disruptive to parent child interactions. Second, our study relies on a relatively homogenous, low risk convenience sample. Replications with families facing higher levels of socioeconomic adversity are warranted. Lastly, in the present study we did not consider work-related screen use, which could additionally contribute to child outcomes. Even though we found prospective associations between parent screen use and preschooler global development, it was not possible to control for baseline measures of child global development or address the possibility of reverse causation since our outcome measurement was only administered at our follow-up assessment. As such, studies using repeated measures of child global development could help clarify the extent to which parent use of screen media may contribute to children’s developmental characteristics above and beyond their baseline global development.

Future studies could shed light on parent characteristics and the nature of their screen use that may contribute to child outcomes. For instance, mothers more often divide their attention between their device and their child, whereas fathers are more likely to remain more continuously focused on their phone (Kiefner-Burmeister et al., 2020). Research could clarify the extent to which additional parent characteristics (ex., mental health) contribute to screen use and child outcomes. The nature of parents’ screen use is also likely to represent an important moderator in the association between their screen habits and their interactions with their child. For instance, studies have found that parents interact more and show more engagement toward children when taking a picture, then when texting or swiping (Bury et al., 2020). Research has found that the most popular activities observed during parent phone use are texting or swiping (43%), looking at the screen (22%), making calls (22%), and taking pictures of the child (Ochoa et al., 2021). As such, research could seek to better understand which parental screen use activities are likely to interfere with ongoing parent-child interactions and those that are least likely to interfere.

To date, most pediatric societies have focused on sensitizing parents to the potential of consequences of excessive screen use by children with little or no attention given to parent screen use. The present findings suggest that parents of young children should be encouraged to limit their screen time to ensure optimal developmental outcomes in early childhood. Our results also suggest that parent-child interactions may be a promising intervention target for reducing or minimizing harms occasioned by family screen use. Intervention efforts could aim to sensitize parents about their own screen habits and their potential impacts on child developmental outcomes. Furthermore, our results suggest that parents should be encouraged to prioritize screen free activities with children like imaginary play, physical activities, and shared book reading to help foster strong global development skills. This remains all the more important in the context of widespread family screen use in the ecology of young children.

## Data availability statement

The data for the present study are not readily available. As per the participant consent form, data are only available to the research team. Any requests for the raw data should be addressed to the coresponding author.

## Ethics statement

The studies involving humans were approved by the Université Sainte-Anne Comité d’Étique. The studies were conducted in accordance with the local legislation and institutional requirements. Written informed consent for participation in this study was provided by the participants’ legal guardians/next of kin.

## Author contributions

CF: Conceptualization, Data curation, Funding acquisition, Methodology, Project administration, Writing – original draft. AJ: Formal analysis, Methodology, Writing – review and editing. AL: Writing – review and editing. MB: Writing – review and editing. EH: Conceptualization, Formal analysis, Supervision, Writing – review and editing.
